# Lifetime classic psychedelic use and headaches: A cross-sectional study

**DOI:** 10.1177/02698811251324372

**Published:** 2025-03-12

**Authors:** Zusanna Bjurenfalk, Alva Cosmo, Otto Simonsson, Caroline Ran

**Affiliations:** 1 Department of Neuroscience, Centre for Cluster Headache, Karolinska Institutet, Stockholm, Sweden; 2 Department of Neurobiology, Care Sciences and Society, Karolinska Institutet, Stockholm, Sweden

**Keywords:** Cluster headache, LSD, migraine, psilocybin, psychedelics

## Abstract

**Background:**

Migraine and cluster headache are two primary headache disorders for which conventional treatments are limited. Classic psychedelic substances such as lysergic acid diethylamide (LSD) and psilocybin are potentially promising new treatment candidates for these conditions.

**Aims:**

The aim of the present study was to investigate the possible relationship between the lifetime use of classic psychedelics and frequent bad headaches in a large British cohort sample.

**Methods:**

Using data (*N* = 11,419) collected in 1999–2000 as part of the 1958 British National Child Development Study, this cross-sectional study used multiple logistic regression, controlling for a range of potential confounders, to test the hypothesis that lifetime use of classic psychedelics would be associated with lower odds of having frequent bad headaches.

**Results:**

Lifetime use of classic psychedelics was associated with 25% lower odds of having frequent bad headaches (adjusted odds ratio = 0.75, 95% CI: 0.59–0.95, *p* = 0.016).

**Conclusions:**

The results of the present study add to the literature suggesting classic psychedelics as a possible future prophylactic treatment option for primary headache disorders.

## Introduction

Migraine and cluster headache are two types of primary headache disorders categorized by head and/or face pain. Migraine is a common headache disorder with an estimated prevalence of 15% and is one of the most disabling diseases worldwide (Vos et al., 2017). Migraine attacks last between 4 and 72 h and are characterized by unilaterally located throbbing or pulsing pain, accompanied by other symptoms such as nausea and high sensitivity to light and/or sound. Migraine is classified either as episodic or chronic depending on the frequency of attacks. Chronic migraine is defined as having 15 or more headache days each month, with at least eight of those days featuring migraine symptoms (International Headache Society, 2018). Cluster headache is a rare but severe headache disorder with an estimated prevalence of 0.05%–0.1% (Fischera et al., 2008; Steinberg et al., 2019). It is marked by recurrent attacks of intense and strictly unilateral orbital, supraorbital, and/or temporal pain accompanied by cranial and/or systemic autonomic symptoms. The headache attacks last 15–180 min and occur once every other day up to eight times a day. Cluster headache may present either in an episodic form, with attacks occurring in periods lasting a few weeks to a few months followed by attack-free remission periods, or in a chronic form when the active periods last 12 months or more with remission periods shorter than 3 months (International Headache Society, 2018). It is considered one of the most painful medical conditions, has been referred to as the “suicide headache,” and has been associated with suicidal ideations (Burish et al., 2021; Ji Lee et al., 2019; Nesbitt and Goadsby, 2012; Rozen and Fishman, 2012). Migraine and cluster headaches are pain disorders that are usually managed with pharmacological treatments to abort attacks (acute treatment) as well as a prophylactic treatment to reduce headache frequency. Conventional treatments are limited in terms of their efficacy and are associated with side effects (Hepp et al., 2015; Lund et al., 2023). Therefore, the need for new treatment paradigms is great.

Preliminary findings from the 1960s suggested that classic psychedelic substances might have a role in treating headache disorders (Kast, 1967; Sicuteri, 1963). Classic psychedelics is a term that refers to a group of psychoactive compounds (e.g., lysergic acid diethylamide (LSD) and psilocybin) that mainly act as serotonin 2A (5-HT2A) receptor agonists. These substances have reportedly since ancient times been used by different cultures worldwide, and they are known to alter perception and induce altered states of consciousness (Nichols, 2016). Over the last few decades, the potential therapeutic effects of classic psychedelics have been investigated for various mental health and chronic pain conditions (Nutt and Carhart-Harris, 2021; Whelan and Johnson, 2018). Risks associated with the use of classic psychedelics are mainly psychological, and there is some potential for abuse (Nichols, 2016). However, the scope of use and associated harms appear to be lower compared to those of prototypical abused drugs (Johnson et al., 2018).

Evidence from modern-day clinical trials suggests there may be a potential preventative effect of classic psychedelics on migraine and cluster headaches (Goel et al., 2023). For example, an exploratory double-blind proof-of-concept study found a significantly greater reduction in migraine symptoms (e.g., migraine attack frequency and pain intensity) 2 weeks after a single dose of psilocybin compared with placebo (Schindler et al., 2021). Another clinical trial, with an open-label study design, found a significant reduction in headache frequency and a reduction in mean self-rated pain 4 weeks after administration of 3 doses of psilocybin over a 3-week period, in patients with chronic cluster headache (Madsen et al., 2024). Yet another trial similarly found a significant reduction in headache frequency 12 weeks after treatment with 3 doses of bromolysergide (BOL-148), a non-hallucinogenic LSD derivative was distributed over a 15-day period (Karst et al., 2010). However, Schindler et al. (2022) found no statistically significant differences in the reduction of cluster headache symptoms between psilocybin and placebo in an exploratory randomized controlled trial of psilocybin for cluster headache (Schindler et al., 2022). The authors speculate that the lack of statistically significant differences may be due to the small sample size (*n* = 14), the differences in effect size between episodic and chronic cluster headache patients, and/or the low dosage. However, in a follow-up on 10 of the patients, 6 months after the first study, the authors reported a significant reduction in attack frequency and pain intensity 3 weeks after initiating another treatment with psilocybin (3 doses over a 15-day period; Schindler et al., 2024). Moreover, studies based on self-reported data have suggested that classic psychedelics may be effective as self-medication for migraine and tension-type headaches (Andersson et al., 2017; Cavarra et al., 2023), and that they may terminate cluster headache periods, prolong remission periods, and have acute effects on headache attacks, in people with cluster headache (Andersson et al., 2017; Schindler et al., 2015; Sewell et al., 2006).

The mechanisms of action of classic psychedelics in headache disorders are currently being investigated. Some reports suggest anti-inflammatory and immunomodulatory mechanisms of action (Flanagan and Nichols, 2018; Thompson and Szabo, 2020). Other hypotheses involve alterations in the melatonin release and/or the secretion of other hormones (Chazot et al., 1984; Kennaway et al., 2001; Leone et al., 1996; Neeb et al., 2015; Phillips et al., 2006; Schmid et al., 2015). In cluster headaches, alterations in the blood flow and functional connectivity of the hypothalamus have been proposed as mechanisms of action (Carhart-Harris et al., 2012; Madsen et al., 2024).

Overall, there is still limited knowledge of the effectiveness of classic psychedelics in primary headache disorders, and existing studies are limited by their small sample size. To increase the power of analysis, one possibility is to utilize large-scale epidemiological data to investigate the use of classic psychedelics among people who report headaches. The present study aims to investigate the possible relationship between classic psychedelic use and headaches using data from a British cohort. Assuming the hypothesis that classic psychedelics may be effective as prophylactic treatment for headache disorders is true, we hypothesize that a concordant relationship may be observed in epidemiological data; specifically, that lifetime use of classic psychedelics will be associated with lower odds of having frequent bad headaches. Cluster headache is more common among males, whereas migraine is more common among females (Allais et al., 2020; Fuensalida-Novo et al., 2020; Lund et al., 2019). Given the sex-related differences in the prevalence of headache disorders, and the fact that substance use of various types is more common among males (Riley et al., 2018), we will also investigate sex differences in the potential associations found.

## Materials and methods

### Data

The hypothesis will be explored by using cross-sectional data from the British National Child Development Study 1958 (NCDS). It is a longitudinal cohort study following people born in 1 week in March 1958 in England, Wales, and Scotland (*n* = 17,634). The data used in the current study is from the sixth data collection of this cohort (*n* = 11,419) which was collected in 1999–2000. Most of the data was collected through interviews whereas questions about drug use were part of a set of questions collected through self-completion on a computer. The data collection was approved by the London multicenter research ethics committee and the participants received an informational letter before the procedure (University of London, 2014). Details about the cohort, the study design, and the data collection are further described elsewhere (Bynner et al., 2002; Power and Elliott, 2006; Shepherd, 2001). Variables used in the analysis are specified in the section below and in the Supplemental material. The data is pseudonymized and available via the UK Data Service (University of London, 2008). The study was approved by the Swedish Ethical Review Authority (Registration Number 2023-07857-02).

### Variables

The dependent variable was headaches measured by the item “Do you often have bad headaches?” (variable mal04; yes or no). The independent variable was the lifetime use of classic psychedelics measured by the items “Have you ever tried LSD, also known as acid or trips?” (variable LSD) and “Have you ever tried magic mushrooms?” (variable magmush). These variables were combined so that “Yes not in last 12 months” or “Yes in last 12 months” in response to either variable was coded as “Yes,” whereas “Never” in response to both variables was coded as “No.” Sensitivity analyses were conducted including only individuals who had used psychedelics within the last 12 months, in the last 12 months and previously, and only those who had used psychedelics before the 12 months preceding the data collection. Sex, marital status, and financial status were included as demographic covariates to describe the nature and distribution of the sample. Considering that headache disorders have been linked to high body mass index and unhealthy lifestyles, we also included physical activity and self-perception of weight as covariates. Given previous reports on associations between headaches and use of different substances (Błaszczyk et al., 2023; Taylor, 2015; Toth, 2003), including substance use for medicinal purposes (Poudel et al., 2021; Ray et al., 2022), we included alcohol, smoking, and lifetime use of cannabis and ketamine as covariates. Finally, we included lifetime use of other illicit drugs as a covariate to account for other substance abuse (Lund et al., 2019; Robberstad et al., 2010; van den Hoek et al., 2024). See Supplemental material for detailed information about the covariates.

### Statistical analyses

All participants with missing data on any of the variables of interest were excluded from the dataset, Supplemental material, Figure S1. Descriptive statistics were calculated using Chi-squared (χ^2^) tests to provide an overview of the sample. Multiple logistic regression modeling with calculated adjusted odds ratios and 95% confidence intervals was used to test the hypothesis and investigate potential sex differences. The alpha level for significance was set at 0.05. Statistical analyses were performed in R studio using R version 4.3.1 (“Beagle Scouts”) and the “tab_model” function in the “sjPlot” package was used to calculate adjusted odds ratios and confidence intervals (R Core Team, 2023).

## Results

### Characteristics of study participants

**Figure 1.**
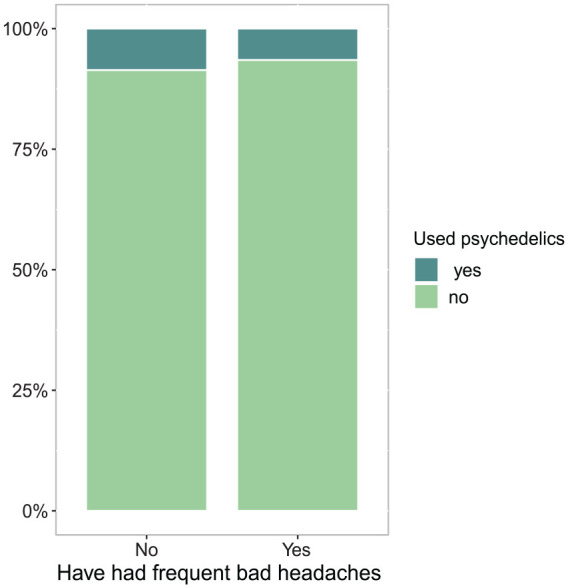
Lifetime use of classic psychedelics among participants with or without frequent bad headaches was reported by 6.5% and 8.6% of the participants respectively.

A number of 139 participants were excluded from the analyses due to missing data on all variables. A number of 29 participants were excluded from the analyses due to missing data on the dependent variable “Do you often have bad headaches” (*n* = 5), the independent variable “Have you ever tried (LSD, magic mushrooms)” (*n* = 4) and/or the control (*n* = 20) variables, leaving 11,251 participants for the analyses (Supplemental material, Figure S1). The prevalence of frequent bad headaches in the sample was 16%. Of the participants reporting having frequent bad headaches, 71% were females and 29% were males. Lifetime use of classic psychedelics was reported by 6.5% (*n* = 120) of the participants with frequent bad headaches and by 8.6% (*n* = 807) of the participants without frequent bad headaches (Figure 1).

The descriptive variables were compared between participants who reported frequent bad headaches and participants who did not (Table 1) and were found to differ in proportion between the two groups for all variables except ketamine use. For example, analysis of the demographic variables showed differences in marital status and financial situation. A smaller proportion of the participants with frequent bad headaches reported engaging in regular physical activity (68% vs 75%, *p* < 0.001), and overweight was more frequently reported in the group with frequent bad headaches. In addition, alcohol consumption differed between groups, and a smaller proportion of the participants with frequent bad headaches reported that they consume/have consumed cannabis (27% vs 31%, *p* = 0.001). Ketamine use in both groups was very limited. However, the proportion of participants who have/had consumed other illicit drugs was larger among those who reported frequent bad headaches (*p* = 0.004).

**Table 1.** Characteristics of the sample based on headache experiences.

Characteristic	Have frequent bad headaches % (*n*)		Group comparisons	
	Yes	No	χ^2^ (*df*)	*p*-Value
Self-rated financial situation				
Living comfortably	30 (552)	38 (3576)	92.80 (4)	<0.001
Doing alright	32 (595)	34 (3204)		
Just about getting by	26 (479)	21 (1935)		
Finding it quite difficult	8.3 (153)	5.2 (489)		
Finding it very difficult	3.7 (69)	2.1 (199)		
Marital status				
Married	70 (1288)	71 (6664)	11.75 (3)	0.008
Divorced/separated	18 (332)	16 (1477)		
Widowed	1 (18)	0.6 (54)		
Never married	11 (210)	13 (1208)		
Self-experienced weight				
Underweight	5.4 (99)	4.5 (419)	80.87 (3)	<0.001
About the right weight	28 (515)	34 (3171)		
Slightly overweight	46 (855)	49 (4585)		
Very overweight	21 (379)	13 (1228)		
Regular physical activity				
Yes	68 (1265)	75 (7087)	38.27 (1)	<0.001
Cigarette smoking				
Never	43 (799)	45 (4216)	16.92 (3)	<0.001
Used to smoke	24 (449)	25 (2391)		
Occasionally	3.2 (60)	4.5 (424)		
Every day	29 (540)	25 (2372)		
Alcohol use				
Never	2.1 (39)	1.2 (115)	296.38 (6)	<0.001
Never now a days	7.8 (145)	3.1 (289)		
On special occasions	21 (379)	12 (1108)		
2–3 times a month	13 (243)	10 (956)		
Once a week	19 (353)	19 (1754)		
2–3 days a week	24 (436)	34 (3213)		
On most days	14 (253)	21 (1968)		
Lifetime cannabis use				
Yes	27 (499)	31 (2903)	10.79 (1)	0.001
Lifetime ketamine use				
Yes	8 (0.4)	30 (0.3)	0.30 (1)	0.580
Lifetime other illicit drug use				
Yes	17 (318)	15 (1375)	7.87 (1)	0.005

Group comparisons were based on having frequent bad headaches (*n* = 1848) or not having frequent bad headaches (*n* = 9403).

χ^2^: Chi-square; *df*: degrees of freedom.

Lifetime use of classic psychedelics was reported by 12% of the males with and 11% of the males without frequent bad headaches, as compared to 4.2% and 5.5% of the females with and without frequent bad headaches. As expected, alcohol and drug use were overall reported more often in males than females (Supplemental material, Table S1).

The characteristics of the sample based on sex and lifetime use of classic psychedelics can be found in Table 2. Lifetime use of classic psychedelics was more common among males (68%) than females (32%), Chi-square (χ^2^) = 140.3, *p* < 0.001. The demographic variables differed significantly in proportion between the groups of lifetime use and non-lifetime use of classic psychedelics. The proportions of participants reporting use of other substances were also significantly higher among lifetime classic psychedelic users than among non-lifetime classic psychedelic users, with approximately 50% versus 28% smoking, 33% versus 19% drinking alcohol on most days, 93% versus 25% reporting lifetime use of cannabis (*p* < 0.001), 3.5% versus <0.1% reporting lifetime use of ketamine (*p* < 0.001), and 70% versus 10% reporting lifetime use of other illicit drugs (*p* < 0.001).

**Table 2.** Characteristics of the sample based on sex and lifetime use of classic psychedelics.

Characteristic	Lifetime classic psychedelic use		Non-lifetime classic psychedelic use		Group comparisons	
	All % (*n*)	Male/female %	All % (*n*)	Male/female %	χ^2^ (*df*)	*p*-Value
Frequent bad headaches (yes)	13 (120)	10/18	17 (1728)	9.8/23	8.64 (1)	0.003
Self-rated financial status						
Living comfortably	32 (293)	33/29	37 (3835)	37/37	43.87 (4)	<0.001
Doing alright	31 (290)	29/35	34 (3509)	34/34		
Just about getting by	24 (224)	25/23	21 (2190)	22/21		
Finding it quite difficult	9.7 (90)	9.9/9.4	5.3 (552)	5.2/5.5		
Finding it very difficult	3.2 (30)	3.2/3.3	2.3 (238)	2.1/2.5		
Marital status						
Married	54 (499)	54/53	72 (7453)	73/72	176.21 (3)	<0.001
Divorced/separated	21 (198)	21/22	16 (1611)	14/17		
Widowed	0.2 (2)	0.2/0.3	0.7 (70)	0.3/1		
Never married	25 (228)	25/24	70 (1190)	13/10		
Self-experienced weight						
Underweight	7.2 (67)	8.8/4	4.4 (451)	5.2/3.6	37.0 (3)	<0.001
About the right weight	38 (348)	38/36	32 (3,338)	33/32		
Slightly overweight	45 (419)	47/42	49 (5021)	52/46		
Very overweight	10 (93)	6.5/17	15 (1514)	9.7/19		
Regular physical activity (yes)	77 (717)	77/78	74 (7635)	75/73	4.94 (1)	0.026
Cigarette smoking						
Never	19 (175)	20/17	47 (4840)	47/47	299.01 (3)	<0.001
Used to smoke	31 (290)	30/33	25 (2550)	26/24		
Occasionally	8.5 (79)	8.4/8.7	3.9 (405)	4,1/3.8		
Every day	41 (383)	41/41	24 (2529)	24/25		
Alcohol use						
Never	0.8 (7)	0.6/1	1.4 (147)	1/1.8	126.32 (6)	<0.001
Never now a days	4.1 (38)	3.7/5.0	3.8 (396)	3/4.6		
On special occasions	8.3 (77)	7.5/10	14 (1410)	8.9/18		
2–3 times a month	7.6 (70)	7.2/8.4	11 (1129)	9/13		
Once a week	15 (141)	14/19	19 (1966)	18/13		
2–3 days a week	31 (289)	32/30	33 (3360)	36/29		
On most days	33 (305)	36/27	19 (1916)	23/14		
Lifetime cannabis use (yes)	93 (864)	93/94	25 (2538)	31/19	1895.5 (1)	<0.001
Lifetime ketamine use (yes)	3.5 (32)	3.8/2.7	<0.1 (6)	<0.1/<0.1	281.08 (1)	<0.001
Lifetime illicit drug use (yes)	70 (649)	71/67	10 (1046)	11/9.3	2382.7 (1)	<0.001

Characteristics of individuals reporting lifetime use of psychedelics (*n* = 927; 628 males and 299 females) or not reporting lifetime use of psychedelics (*n* = 10,324; 4895 males and 5429 females) based on the whole group (% and numbers) and based on sex (%). Group comparisons were based on use of classic psychedelics in the whole cohort.

χ^2^: Chi-square; *df*: degrees of freedom.

### Use of classic psychedelics among participants with frequent bad headaches

The results of the multiple logistic regression modeling for the associations between lifetime use of classic psychedelics and frequent bad headaches are shown in Table 3. In unadjusted analyses, lifetime use of classic psychedelics was associated with 26% lower odds of having frequent bad headaches (OR = 0.74, 95% CI: 0.60–0.90, *p* = 0.003). In covariate-adjusted analyses, lifetime use of classic psychedelics was associated with 25% lower odds of having frequent bad headaches (aOR = 0.75, 95% CI: 0.59–0.95, *p* = 0.016). Several covariates were associated with frequent bad headaches (Supplemental material, Table S2). Last, sensitivity analyses were run including only individuals who had used classic psychedelics during the last 12 months, in the last 12 months and previously, or in the past but not during the last 12 months. The first analysis, including only current users, yielded non-significant results, probably because of the small sample size and lack of statistical power. The other two analyses confirmed the association in both current and past users of psychedelic drugs, Supplemental material, Table S3. In unadjusted analyses performed in males and females separately, no association was found between lifetime use of classic psychedelics and frequent bad headaches in males (OR = 1.06, 95% CI: 0.80–1.39), *p* = 0.655) or in females (OR = 0.75, 95% CI: 0.55–1.01, *p* = 0.065). In covariate-adjusted analyses performed in males and females separately, no association was found between lifetime use of classic psychedelics and frequent bad headaches in males (aOR = 0.74, 95% CI: 0.53–1.02, *p* = 0.073), whereas in females, lifetime use of classic psychedelics was associated with 30% reduced odds of having frequent bad headaches (aOR = 0.70, 95% CI: 0.49–0.98, *p* = 0.039). Further statistical analysis did not reveal any interaction between psychedelic use and sex affecting the odds of having frequent bad headaches (*p* = 0.096).

**Table 3.** Association between lifetime use of classic psychedelics and frequent bad headaches.

Variables	Frequent bad headaches					
	OR	95% CI	*p*-Value	aOR	95% CI	*p*-Value
Lifetime classic psychedelic use	0.74	0.60–0.90	0.003	0.75	0.58–0.94	0.013
Lifetime classic psychedelic use in males	1.06	0.80–1.39	0.655	0.73	0.52–1.01	0.062
Lifetime classic psychedelic use in females	0.75	0.55–1.01	0.065	0.70	0.49–0.98	0.039

The association between lifetime use of classic psychedelics and frequent bad headaches was analyzed using multiple logistic regression. aORs were adjusted for sex, self-rated financial situation, marital status, regular physical activity, self-experienced weight, cigarette smoking, alcohol use, lifetime use of cannabis, ketamine, and lifetime use of other illicit drugs.

OR: unadjusted odds ratio; CI: confidence interval; aOR: adjusted odds ratio.

## Discussion

Migraine and cluster headache are two types of primary headache disorders for which conventional treatments are limited in terms of their efficacy and their association with side effects (Hepp et al., 2015; Lund et al., 2023). Classic psychedelics are potentially promising as a treatment option for several chronic pain conditions, including migraine and cluster headache (Goel et al., 2023). The main objective of this cross-sectional study was to investigate the possible association between lifetime classic psychedelic use and headaches using a British cohort sample. We found that lifetime use of classic psychedelics was associated with lower odds of reporting frequent bad headaches. This is consistent with our hypothesis and adds to the literature suggesting a potential benefit of classic psychedelics in headache disorders (Goel et al., 2023).

Comparing classic psychedelics to conventional drugs used in the treatment of headache disorders supports the role of psychedelics in the treatment of headache disorders. There are chemical and pharmacological similarities between LSD, psilocybin, and conventional drugs used in the treatment of migraine (Kooijman et al., 2023). The first drug used as a prophylactic treatment for migraine was daily doses of methysergide (Sewell, 2008). Methysergide acts on many serotonin receptors, it differs slightly from LSD structurally and is a potent 5-HT2A receptor antagonist. Unfortunately, this medication was found to have severe side effects and has therefore been removed from the US market (Sewell, 2008). A commonly used drug for acute treatment of migraine attacks is dihydroergotamine, a 5-HT2A receptor agonist. However, it is proposed that the abortive effect of dihydroergotamine is mediated by its receptor agonist activity of the serotonin receptor 1B (5-HT1B) and 1D (5-HT1D) (Silberstein and McCrory, 2003). Similarly, serotonin receptors 5-HT1B and 5-HT1D are the primary binding site of triptans, the first-line acute treatment for both migraine and cluster headache.

Headache disorders have previously been associated with a higher prevalence of smoking, illicit drug use, high body mass index, and lower levels of physical activity compared to the general population (Lund et al., 2019; Robberstad et al., 2010). However, another study found the opposite association where migraine patients were less likely to smoke, drink alcohol, or use illicit drugs compared to controls (van den Hoek et al., 2024). Similarly to previous reports, we found that low alcohol use was associated with higher odds of having frequent bad headaches. This finding is in line with alcohol being a trigger for headaches; the observed positive association between low alcohol use and frequent bad headaches may be explained by individuals experiencing frequent bad headaches choosing to abstain from alcohol (Hindiyeh et al., 2020; Toth, 2003). The proportion of participants reporting to have used cannabis was also lower in the headache group. Conversely, lifetime use of illicit drugs was associated with higher odds of having frequent bad headaches in our study. There might be several reasons behind the conflicting data on illicit drugs. First, our analysis highlights the importance of the choice of statistical model. We found an association in opposite directions for lifetime use of illicit drugs and lifetime use of classic psychedelics with having frequent bad headaches, implying the association might be overlooked if these substances are analyzed together. Second, alcohol and various types of illicit drugs have been associated with migraine and other types of headaches and might also cause headaches by themselves, which are then defined as secondary headaches (Hindiyeh et al., 2020; Johnson et al., 2012; Toth, 2003). This is an effect that we might be overlooking in this dataset because of the lack of clinical diagnoses for specific types of headaches, which implies that individuals with secondary headaches are included in the analysis.

Subgroup analysis revealed a significant association between lifetime use of classic psychedelics and frequent bad headaches only in females while a larger proportion of the males in our sample reported daily alcohol use and lifetime use of other illicit drugs. This is consistent with previous reports on sex differences in drug and alcohol use (Riley et al., 2018). The higher use of different substances among the male participants in our sample may further indicate an overall less healthy lifestyle. This is a factor that has also been associated with recurrent headaches (Robberstad et al., 2010), and is also modeled in our study by the variables weight and physical exercise. Being very overweight and lack of regular physical activity was indeed more common in study participants with headaches also in our study, but not particularly in male participants. We hypothesize that a possible association between lifetime classic psychedelic use and lower odds of headache in males is masked by a disproportionately elevated drug use in combination with a smaller sample size in the male strata, reflecting the lower incidence of headache in the male population. In this cohort, only 179 males reported lifetime use of psychedelic drugs without having used other illicit drugs as well (cannabis exempted). In this small subgroup, the proportion of participants with frequent bad headaches was smaller (5.6%, *n* = 10) than in the group of male participants who had never used psychedelic drugs or other drugs (9.1%, *n* = 398). These numbers support our hypothesis but need to be confirmed in a larger cohort before we can draw conclusions. It is also possible that the differences we observe in male and female subgroups are the result of biological differences in the response to psychedelics. Little is known about sex differences in the physiological response to psychedelics in humans, but data from animal models suggest the topic of sex differences in psychedelics is worth investigating further. Behavioral differences between male and female rodents have been observed in response to psychedelics in experimental settings, as well as differences at a cellular (dendritic spine density) and molecular level (gene expression), (Shadani et al., 2024).

The present study has some limitations. The observational nature of the study is a major limitation. Although we have proposed a direction of association, we cannot draw any causal inferences about the association between lifetime use of classic psychedelics and frequent bad headaches. It is possible that the negative association found is a result of people suffering from frequent bad headaches abstaining from the use of classic psychedelics. This could be plausible, considering that acute headache is a common adverse effect of the use of classic psychedelics (Johnson et al., 2012). However, the proportion of people with frequent bad headaches who used other illicit drugs was slightly higher than in the group without frequent bad headaches, particularly in males. In addition, we do not have information on behaviors typically associated with the use of illicit drugs, including psychedelic substances, such as abuse, having experienced stressful life events or adverse childhood experiences, or any other latent variable that may influence the dependent variable, which we have not included in this analysis. Another limitation relates to the items that were used. As the data were self-reported, participants may have interpreted the questions and reported their answers in different ways. This is particularly important with regard to the variable “bad headache.” Due to the wording of the item “Do you often have bad headaches?,” there is no information on whether the participants had a headache diagnosis or on the intensity or frequency of their symptoms. Therefore, it is not possible to generalize our results to a clinical population. The statistical model implies both strengths and limitations. Using a logistic regression to assess the associations between often having bad headaches and lifetime use of classic psychedelics is efficient and the outcome variables indicate coefficient size and direction of the associations and are easy to interpret. On the other hand, logistic regression assumes linearity of the variables and nonlinear complex interactions may not be properly captured. Furthermore, the model is based on the independence of variables, which is often not the case with observational data and may influence the results. A strength of the study is the large and comparatively unbiased sample. A common problem in the classic psychedelic research field is bias in the selection of participants. It is reasonable to suspect that studies explicitly investigating classic psychedelics are likely to attract people who have a prior interest in classic psychedelics and positive beliefs about their effects on mental or physical health. However, the sample used in the present study was recruited at birth and did not volunteer on the basis of the research topic. Furthermore, the NCDS was designed to collect data on a range of topics affecting human development across the lifespan, and the section on drug use was only a small part of the survey.

## Conclusion

Classic psychedelic substances and their potential therapeutic effects are currently being investigated. Our finding of a negative association between lifetime use of classic psychedelics and frequent bad headaches adds to the literature suggesting that these substances may have therapeutic effects on headache disorders. Future research should continue to investigate the potential prophylactic effects and possible mechanisms of action of classic psychedelics in headache disorders, such as migraine and cluster headaches.

## Supplemental Material

sj-docx-1-jop-10.1177_02698811251324372 – Supplemental material for Lifetime classic psychedelic use and headaches: A cross-sectional study Supplemental material, sj-docx-1-jop-10.1177_02698811251324372 for Lifetime classic psychedelic use and headaches: A cross-sectional study by Zusanna Bjurenfalk, Alva Cosmo, Otto Simonsson and Caroline Ran in Journal of Psychopharmacology
