# Supplementary material for: Lifetime classic psychedelic use and headaches: A cross-sectional study
Source: J Psychopharmacol. 2025 Mar 12;39(9):968–75. doi: 10.1177/02698811251324372 (PMC12371135; doi:10.1177/02698811251324372)
Supplement: sj-docx-1-jop-10.1177_02698811251324372 – Supplemental material for Lifetime classic psychedelic use and headaches: A cross-sectional study [file sj-docx-1-jop-10.1177_02698811251324372.docx]

**Supplementary material**

**Contents**

[Information about the covariates 2](#_Toc187596483)

[Figure S1. Flow diagram showing exclusion of study participants. 3](#_Toc187596484)

[Table S1. Characteristics of the sample based on sex and headache experiences. 4](#_Toc187596485)

[Table S2. Covariate-adjusted logistic regression model for the association between lifetime use of classic psychedelics and frequent bad headaches. 5](#_Toc187596486)

[Table S3. Sensitivity analyses for current and past use of classic psychedelics. 6](#_Toc187596487)

# **Information about the covariates**

The following covariates were included in the adjusted analyses:

**Sex** (variable n622_6; male or female).

**Marital status** (variable marstat2; ‘What is your current, legal marital status?’; where ‘married - 1st & only marriage’ and ‘remarried - 2nd or later marriage’ were coded as ‘married’, ‘legally separated and ‘divorced’ were coded as ‘divorced/separated’, ‘single never married’ was coded as ‘single’, whereas ‘widowed’ was not recoded).

**Financial status** (variable finnow; ‘How well would you say you yourself are managing financially these days?’; financially stable, doing alright, living comfortably, finding it quite difficult, just about getting by or finding it very difficult).

**Physical activity** (variable exercise; ‘Do you regularly [at least once a month, for most of the year] take part in any of the activities on this card?’; yes or no).

**Perception of own weight** (variable wtassess; ‘Would you say you were ‘about the right weight’, ‘underweight’, ‘slightly overweight’ or ‘very overweight’?’; about the right weight, slightly overweight, very overweight or underweight).

**Smoking habits** (variable smoking; smoke cigarettes occasionally, smoke cigarettes every day, used to smoke but don’t at all now or never smoked cigarettes).

**Alcohol use** (variable drinks; ‘How often do you have an alcoholic drink of any kind?’, on most days, 2 to 3 days a week, once a week, 2 to 3 times a month, only on special occasions, never now a days or never had an alcoholic drink).

**Lifetime cannabis use** (variable cannabis; ‘Have you ever tried cannabis, also known as blow, draw, puff, grass, skunk, weed, black, hash or red seal?’; where ‘Yes not in last12mths’ and ‘Yes in last 12mths’ were coded as ‘Yes’ whereas ‘Never’ was coded as ‘No’).

Lifetime ketamine use (variable ketamine; ‘Have you ever tried ketamine?’; where ‘Yes not in last12mths’ and ‘Yes in last 12mths’ were coded as ‘Yes’ whereas ‘Never’ was coded as ‘No’).

**Lifetime use of other illicit drugs** (variables ecsasy, amphet, popper, cocaine, temaz, crack, heroin, methad, othdrug; measured by ‘Have you ever tried...’ followed by the following substances and their nicknames: ‘ecstasy’, ‘amphetamines’, ‘amyl nitrate’, ‘cocaine’, ‘temazepam’, ‘crack’, ‘heroin’, ‘methadone’ or ‘any other illegal drugs not mentioned earlier’; where ‘Yes not in last12mths’ and ‘Yes in last 12mths’ in response to any of the items were coded as ‘Yes’ whereas ‘Never’ in response to all of the items was coded as ‘No’).

# **Figure S1. Flow diagram showing exclusion of study participants.**

**Missing data comprises a) an entry with a missing value “NA”, b) the study participant not answering the question ”Not answered” and c) the study participant having answered ”Do not know”.*

# **Table S1. Characteristics of the sample based on sex and headache experiences.**

| **Characteristic** | | **Have frequent bad headaches % (n)** | | **Doesn’t have frequent bad headaches % (n)** | |
| --- | --- | --- | --- | --- | --- |
|  | | **Males**  **(n = 544)** | **Females**  **(n = 1,304)** | **Males**  **(n = 4,979)** | **Females**  **(n = 4,424)** |
| Self-rated financial situation | Living comfortably | 25 (135) | 32 (417) | 38 (1,900) | 38 (1,676) |
|  | Doing alright | 31 (166) | 33 (429) | 33 (1,659) | 35 (1,545) |
|  | Just about getting by | 29 (160) | 24 (319) | 21 (1,062) | 20 (873) |
|  | Finding it quite difficult | 10 (57) | 7.4 (96) | 5.2 (261) | 5.2 (228) |
|  | Finding it very difficult | 4.8 (26) | 3.3 (43) | 1.9 (97) | 2.3 (102) |
| Marital status | Married | 68 (372) | 70 (916) | 71 (3,519) | 71 (3,145) |
|  | Divorced/Separated | 16 (88) | 19 (244) | 15 (725) | 17 (752) |
|  | Widowed | 0.7 (4) | 1.1 (14) | 0.2 (12) | 0.9 (42) |
|  | Never married | 15 (80) | 10 (130) | 15 (723) | 11 (485) |
| Self-experienced weight | Underweight | 6.3 (34) | 5 (65) | 5.5 (275) | 3.3 (144) |
|  | About the right weight | 31 (166) | 27 (349) | 34 (1,697) | 33 (1,474) |
|  | Slightly overweight | 50 (273) | 45 (582) | 51 (2,563) | 46 (2,022) |
|  | Very overweight | 13 (71) | 24 (308) | 8.9 (444) | 18 (784) |
| Regular physical activity (yes) | | 68 (372) | 68 (839) | 76 (3,804) | 74 (3283) |
| Cigarette smoking | Never | 36 (196) | 46 (603) | 45 (2,217) | 45 (1,999) |
|  | Used to smoke | 28 (150) | 23 (299) | 26 (1,290) | 25 (1,101) |
|  | Occasionally | 3.7 (20) | 3.1 (40) | 4.7 (233) | 4.3 (191) |
|  | Every day | 33 (178) | 28 (362) | 25 (1,239) | 26 (1,133) |
| Alcohol use | Never | 1.7 (9) | 2.3 (30) | 0.9 (44) | 1.6 (71) |
|  | Never now a days | 6.8 (37) | 8.3 (108) | 2.7 (133) | 3.5 (156) |
|  | On special occasions | 15 (83) | 23 (296) | 8 (398) | 16 (710) |
|  | 2 to 3 times a month | 10 (56) | 14 (187) | 8.6 (430) | 12 (526) |
|  | Once a week | 18 (99) | 19 (254) | 18 (889) | 20 (865) |
|  | 2 to 3 days a week | 26 (143) | 22 (293) | 31 (1,835) | 31 (1,378) |
|  | On most days | 22 (117) | 10 (136) | 25 (1,250) | 16 (718) |
| Lifetime cannabis use (yes) | | 39 (211) | 22 (288) | 38 (1,868) | 23 (1,035) |
| Lifetime ketamine use (yes) | | 5 (0.9) | 3 (0.2) | 22 (0.4) | 8 (0.2) |
| Lifetime other illicit drug use (yes) | | 25 (137) | 14 (182) | 17 (852) | 12 (524) |

# **Table S2. Covariate-adjusted logistic regression model for the association between lifetime use of classic psychedelics and frequent bad headaches.**

| **Variable**  **(reference value)** |  | **Bad Headaches** | | |
| --- | --- | --- | --- | --- |
|  |  | **OR** | **95 % CI** | ***p-value*** |
| (Intercept) |  | 0.07 | 0.05 – 0.09 | **<0.001** |
| Psychedelics (No) | Yes | 0.74 | 0.58 – 0.94 | **0.013** |
| Sex (Male) | Female | 2.41 | 2.16 – 2.70 | **<0.001** |
| Self-rated financial situation  (Living comfortably) | Doing alright | 1.14 | 1.00 – 1.30 | **0.049** |
|  | Just about getting by | 1.45 | 1.26 – 1.67 | **<0.001** |
|  | Finding it quite difficult | 1.81 | 1.46 – 2.25 | **<0.001** |
|  | Finding it very difficult | 1.68 | 1.22 – 2.28 | **0.001** |
| Marital status (Divorced/Separated) | Married | 1.05 | 0.91 – 1.22 | 0.498 |
|  | Single | 0.89 | 0.73 – 1.09 | 0.267 |
|  | Widowed | 1.36 | 0.76 – 2.35 | 0.282 |
| Self-experienced weight (About the right weight) | Underweight | 1.37 | 1.06 – 1.75 | **0.014** |
|  | Slightly overweight | 1.15 | 1.02 – 1.30 | **0.025** |
|  | Very overweight | 1.42 | 1.22 – 1.66 | **<0.001** |
| Alcohol use  (On most days) | 2 to 3 days a week | 1.00 | 0.85 – 1.19 | 0.978 |
|  | Once a week | 1.34 | 1.12 – 1.61 | **0.001** |
|  | 2 to 3 times a month | 1.59 | 1.31 – 1.95 | **<0.001** |
|  | Less often/only on special occasions | 1.88 | 1.56 – 2.26 | **<0.001** |
|  | Never nowadays | 2.94 | 2.29 – 3.76 | **<0.001** |
|  | Never had an alcoholic drink | 1.92 | 1.27 – 2.84 | **0.001** |
| Smoking (Never) | Used to smoke but don't at all now | 1.00 | 0.87 – 1.14 | 0.972 |
|  | Smoke cigarettes occasionally | 0.80 | 0.59 – 1.07 | 0.140 |
|  | Smoke cigarettes every day | 1.02 | 0.89 – 1.17 | 0.760 |
| Regular physical activity (No) | Yes | 0.83 | 0.74 – 0.93 | **0.002** |
| Lifetime cannabis use (No) | Yes | 0.94 | 0.82 – 1.08 | 0.380 |
| Lifetime ketamine use (No) | Yes | 1.40 | 0.57-3.06 | 0.432 |
| Lifetime other illicit drug use (No) | Yes | 1.58 | 1.34 – 1.86 | **<0.001** |

*Results from the logistic regression model* *OR regarding the covariates used. OR: odds ratio; CI: confidence interval; R^2^ Tjur: 0.062; Odds ratios were adjusted for sex, self-rated financial situation, marital status, regular physical activity, self-experienced weight, cigarette smoking, alcohol use, lifetime use of cannabis, ketamine and lifetime use of other illicit drugs.*

# **Table S3. Sensitivity analyses for current and past use of classic psychedelics.**

| **Variables** | **Frequent Bad Headaches** | | **Covariates** | **^a^OR** | **95% CI** | **p-value** |
| --- | --- | --- | --- | --- | --- | --- |
|  | **Yes** | **No** |  |  |  |  |
| Classic Psychedelic use only during last 12 months | 6  (0.3%) | 18 (0.2%) |  | 1.58 | 0.52-4.15 | 0.378 |
| Never used psychedelics | 1728 (99.7%) | 8596 (99.8%) |  |  |  |  |
|  |  |  | Female sex | 2.47 | 2.20-2.78 | **<0.001** |
|  |  |  | Cannabis use | 0.96 | 0.83-1.10 | 0.541 |
|  |  |  | Ketamine use | 2.56 | 0.59-10.51 | 0.186 |
|  |  |  | Use of other drugs | 1.53 | 1.28-1.82 | **<0.001** |
| Classic Psychedelic use during last 12 months and before | 46 (2.6%) | 305 (3.4%) |  | 0.66 | 0.45-0.95 | **0.028** |
| Never used psychedelics | 1728 (97.4%) | 8596 (96.6%) |  |  |  |  |
|  |  |  | Female sex | 2.44 | 2.17-2.74 | **<0.001** |
|  |  |  | Cannabis use | 0.95 | 0.83-1.10 | 0.512 |
|  |  |  | Ketamine use | 1.68 | 0.67-3.85 | 0.243 |
|  |  |  | Use of other drugs | 1.55 | 1.30-1.84 | **<0.001** |
| Classic psychedelic use before but not in the last 12 months | 112 (6.1%) | 774 (8.3%) |  | 0.72 | 0.56–0.91 | **0.007** |
| Never used psychedelics | 1,728 (94%) | 8,596 (92%) |  |  |  |  |
|  |  |  | Female sex | 2.42 | 2.16–2.72 | **<0.001** |
|  |  |  | Cannabis use | 0.95 | 0.82–1.09 | 0.447 |
|  |  |  | Ketamine use | 1.87 | 0.74–4.30 | 0.159 |
|  |  |  | Use of other drugs | 1.56 | 1.32–1.84 | **<0.001** |

*The association between use of classic psychedelics during different time periods and frequent bad headaches were analyzed using multiple logistic regression. ^a^OR: adjusted odds ratio; CI: confidence interval. ^a^ORs were adjusted for sex, self-rated financial situation, marital status, regular physical activity, self-experienced weight, cigarette smoking, alcohol use, lifetime use of cannabis, ketamine and lifetime use of other illicit drugs.*
